# Microhardness, Young’s and Shear Modulus in Tetrahedrally Bonded Novel II-Oxides and III-Nitrides

**DOI:** 10.3390/ma18030494

**Published:** 2025-01-22

**Authors:** Devki N. Talwar, Piotr Becla

**Affiliations:** 1 Department of Physics, University of North Florida, 1 UNF Drive, Jacksonville, FL 32224-7699, USA; 2 Department of Physics, Indiana University of Pennsylvania, 975 Oakland Avenue, 56 Weyandt Hall, Indiana, PA 15705-1087, USA; 3 Department of Materials Science and Engineering, Massachusetts Institute of Technology, Cambridge, MA 02139, USA; becla@mit.edu

**Keywords:** novel II-Os and III-Ns, microhardness, shear modulus, Young’s modulus, bond-orbital and valence force field model, Born’s transverse effective charge

## Abstract

Direct wide-bandgap III-Ns and II-Os have recently gained considerable attention due to their unique electrical and chemical properties. These novel semiconductors are being explored to design short-wavelength light-emitting diodes, sensors/biosensors, photodetectors for integration into flexible transparent nanoelectronics/photonics to achieve high-power radio-frequency modules, and heat-resistant optical switches for communication networks. Knowledge of the elastic constants structural and mechanical properties has played crucial roles both in the basic understanding and assessing materials’ use in thermal management applications. In the absence of experimental structural, elastic constants, and mechanical traits, many theoretical simulations have yielded inconsistent results. This work aims to investigate the basic characteristics of tetrahedrally coordinated, partially ionic BeO, MgO, ZnO, and CdO, and partially covalent BN, AlN, GaN, and InN materials. By incorporating a bond-orbital and a valance force field model, we have reported comparative results of our systematic calculations for the bond length d, bond polarity $\alpha_{P}$, covalency $\alpha_{C}$, bulk modulus B, elastic stiffness $C(=\frac{\left[c_{11}-c_{12}\right]}{2})$, bond-stretching α and bond-bending β force constants, Kleinmann’s internal displacement ζ, and Born’s transverse effective charge $e_{T}^{*}$. Correlations between C/B, β/α, $\frac{c_{12}}{c_{11}},$ ζ, and $\alpha_{C}\;$revealed valuable trends of structural, elastic, and bonding characteristics. The study noticed AlN and GaN (MgO and ZnO) showing nearly comparable features, while BN (BeO) is much harder compared to InN (CdO) material, with drastically softer bonding. Calculations of microhardness H, shear modulus G, and Young’s modulus Y have predicted BN (BeO) satisfying a criterion of super hardness. III-Ns (II-Os) could be vital in electronics, aerospace, defense, nuclear reactors, and automotive industries, providing integrity and performance at high temperature in high-power applications, ranging from heat sinks to electronic substrates to insulators in high-power devices.

## 1. Introduction

In technological development, the search for new materials with improved structural, electrical, elastic, and optical properties has been and still is the motive of many scientists and engineers. Over the last few decades, the use of group III-nitrides (BN, AlN, GaN, and InN) has demonstrated the designing of many short-wavelength electroluminescent devices for electronic and optical data storage needs [1,2]. Strongly distinguished mixed ionic–covalent bonding of III-Ns with extreme hardness, wide bandgaps (WBG) $E_{g}\;$(≡0.7–6.2 eV) and higher thermal conductivity have also offered their potential use in high-temperature, high-power, and high-frequency electronics [3,4,5,6,7,8,9,10,11,12].

In the selection process for applications, researchers have always preferred those elements in materials that are earth abundant and low toxicity [13,14,15,16,17,18]. Oxygen (O), being an important member of group VIA of the periodic table, can form oxides with many metal elements that remain stable in ambient conditions (i.e., temperature T, and pressure P). Combining group IIB elements X (≡Be, Mg, Zn, and Cd) with O has created partially ionic compound semiconductors, which are often classified as II-oxides (or II-Os). Considerable attention has been focused recently on preparing the extremely large WBG $E_{g}\;$(≡2.3–10.7 eV) binary XOs, as well as their ternary and quaternary alloys [19,20,21,22,23,24,25,26]. These materials have potential in developing the advanced electro-optic nanodevices for flexible/transparent electronics and high-power radio-frequency applications. Novel II-oxides are much safer than many other II–VI compounds involving the chalcogens (viz., S, Se, and Te) or III–V semiconductors with pnictogens (i.e., P, As, and Sb). The low cost, environmentally benign, and good crystalline quality of such materials have played important roles in designing functional materials [13,14,15,16,17,18]. Successful epitaxial growth of ultrathin binary and ternary alloys as well as their heterostructures (e.g., multi-quantum wells (MQWs) and superlattices (SLs)) has further stimulated the interest in engineering next-generation device structures to complement the extensively used III-N-based electronic and photonic devices.

One must note that the members of metastable II-Os are partially ionic and exhibit a few similarities with the partially covalent III-Ns (i.e., BeO → BN; MgO, (ZnO, CdO) → AlN, (GaN, InN)). Like carbides, the metal oxides and nitrides are considered hard materials possessing crystal structures, which do not have sufficient slip systems for dislocation movement during the plastic deformations [19,20,21,22,23,24,25,26]. One must note that BeO (BN) exhibits (i) an exceptional hardness, large bond strength, and a high melting point ~2570 °C (~2973 °C), (ii) it is chemically inert to most acids and alkalis, offering excellent corrosion resistance to acids, solvents, and even molten metals, (iii) its thermal conductivity at room temperature (RT) could be as high as ~330 W/mK (~751 W/mK), making it one of the best heat-conducting materials among ceramics, (iv) the BeO (BN) material can be effective as an electronic substrate and insulator, with dielectric strength of approximately 10–16 kV/mm (40 kV/mm), capable of withstanding high-voltage applications without breakdown, (v) it is equally good for rapid heat dissipation to maintain performance and longevity of electronic components. Thus, BeO (BN) can play a crucial role for efficiently managing high temperature variations to ensure operational stability and sustaining efficiency of various electronic devices.

Despite the successful epitaxial growth efforts made in preparing different device structures by incorporating thin films, many fundamental issues of these materials have remained currently unresolved [27,28,29,30,31,32,33,34,35]. For instance, the structural, electronic, elastic, and vibrational properties of II-Os are not thoroughly investigated. To simulate the structural, electronic, and mechanical characteristics, the roles of semi-core cation d-states of O and N in II-Os and III-Ns have indicated playing important roles [27,28]. Besides binary thin films, the use of alloy epilayers for creating a variety of configurations in the layered MQWs and SLs has given endless flexibility of designing and fabricating different electro-optic devices. For III-Ns and II-Os, it is important to explore experimentally and/or theoretically to assess the elastic and structural properties by comparing their basic features based on the physics and chemistry of chemical bonding.

Experimental measurements of the elastic constants $c_{ij}$ are usually performed on relatively large-size single crystals by monitoring either the ultrasonic velocities [36,37] as a function of the external static pressure and/or using Brillouin scattering [38,39]. As III-N and II-O materials have not been grown into large-size samples, the accurate values of their $c_{ij}$ are not available. Earlier attempts made for determining the elastic constants of wurtzite (wz) III-Ns used X-ray measurements on powders and/or poor-quality samples [40,41]. The values of $c_{ij}$ for wz materials were employed for obtaining the $c_{ij}$ for the zinc-blende (zb) GaN and InN with appropriate rotations of the elasticity tensors [32]. A few measurements of $c_{ij}$ are available for the zb AlN, GaN, and BN [33,34] by monitoring sound velocities. However, similar studies [33,34] do not exist for the elastic constants of zb InN. Again, no systematic measurements were performed for assessing the Young’s modulus Y, microhardness H, and bulk modulus B in III-Ns and II-Os.

In the absence of experimental data, many scientists have devoted themselves to developing theoretical tools for effectively synthesizing and/or predicting the structural, electrical, and dynamical traits of these novel materials. Efforts to find trends correlating the bulk, shear, and Young’s moduli with microhardness have received considerable attention [42,43,44,45,46,47,48]. Results based on different first-principles plane-wave pseudopotential methods in the local-density approximation (LDA) are available using the commercial ABINIT software package [49,50,51,52,53,54,55,56,57,58,59]. Linear combination of atomic orbitals (LCAO) [50,51], linear muffin-tin orbitals (LMTO) [53], universal tight-binding parameters (UTBP) [54], molecular dynamics (MD) [55,56], and the extended Hückel tight-binding (XHTB) methods [60,61,62,63,64,65,66] have also been considered. While the ab initio LDA method is known to be an effective and useful tool for studying the structural and electronic properties, the inclusion of total energy in simulating the vibrational and elastic properties has made it rather complicated compared to the empirical valence force field (VFF) [67] and bond-orbital models (BOMs) [60,61,62,63,64,65,66]. Moreover, the published data on the structural, vibrational, and elastic traits using the first-principles methods for III-Ns and II-Os are either inconsistent or questionable [30,31,32,33,34,35,49,50,51,52,53,54,55,56,57,58,59].

The purpose of this work is to adopt a simple tight-binding approach [62,63,64,65,66] (cf. Section 2) and report the results of systematic simulations for the structural and elastic properties (cf. Section 2.1) of novel III-Ns (BN, AN, GaN, and InN) and II-Os (BeO, MgO, ZnO, and CdO). For the binary nitrides, the method (cf. Section 3) predicted bond lengths, d, to an accuracy of within a few percent (viz., BN 0.6%, AlN 1.5%, GaN 2.5%, and InN 5.7%) to the experimental data. In oxides, however, the agreement between limited experimental and/or first-principles calculations of d was moderate (BeO 11.5%, MgO 4.95%, ZnO 12.0%, and CdO 5.4%). Results of the structural and elastic properties (cf. Section 3) are also reported by combining the BOM [62,63,64,65,66] with the VFF [67] scheme. Calculations are performed to predict the bond polarity $\alpha_{p}$, covalency $\alpha_{c}$, stretching force constant α, bond-bending force constant β, elastic constants $c_{11},c_{12},$ and $c_{44}$, bulk moduli B, Poisson’s ratio ν, Kleinman’s internal displacement parameter ζ, Young’s modulus Y, microhardness H, shear modulus G, and effective transverse charge $e_{T}^{*}$, etc. Our calculations provided confirmations to Born’s criteria with the necessary and sufficient conditions $\left(i.e.,\;(c_{11}-c_{12}\right)$ > 0, $\left(c_{11}+2\;c_{12}\right)$ > 0, and $c_{44}$ > 0) that all the materials are mechanically stable. Considering simple arguments based on the physics of chemical bonds, we have attempted possible links between G, Y, H, and the bond lengths d. Only BN (BeO) indicated satisfying the norms of being a super-hard (H > 4 (10^11^ dyn/cm^2^)) material—the other compounds, AlN and GaN (MgO and ZnO), revealed nearly identical but strong values of H (Y,G), as compared to the weak H (Y,G) for InN (CdO) crystal with a longer d. Consistent with the published results from ab initio simulations, the Poisson’s ratio for BN (BeO) was confirmed to have the lower value compared to the other materials. Unlike many complicated empirical relations [49,50,51,52,53,54,55,56,57,58,59], our study for III-Ns and II-Os has provided a linear relationship between the microhardness H and shear modulus G, as well as G and Y, with proportionality constants of ~0.16 and ~0.41, respectively. Theoretical results of different structural, elastic, and mechanical properties are compared/contrasted in Section 3 against the existing data from the first-principles simulations and/or experiments [62,63,64,65,66,67,68] wherever available, with the concluding remarks presented in Section 4. Unlike many sophisticated models, our BOM and VFF study has provided valuable structural, elastic, and mechanical data on III-Ns and II-Os. These results, we hope, will encourage the experimentalists to perform similar measurements to check our theoretical conjectures.

## 2. Background

### 2.1. The Crystal Structures

**Figure 1:**
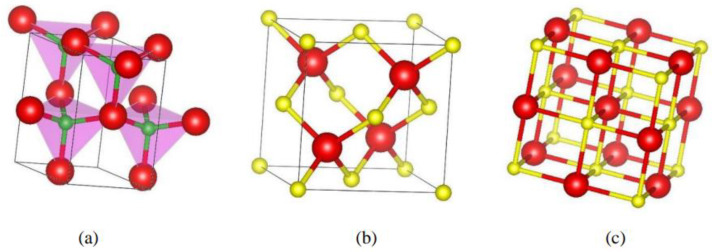
The stick-and-ball representation of different ZnO crystal structures: (**a**) the hexagonal wurtzite (B_4_), (**b**) the cubic zinc-blende (B_3_), and (**c**) the cubic rock salt (B_1_) in the Strukturbericht designation. Small-size green (in (**a**)) and gold (in (**b**,**c**)) colored balls represent the O atoms, while the large-size red-colored spheres signify the Zn atoms.

Like III-Ns, the binary II-Os are known to exist in three different crystalline phases, shown schematically in Figure 1a,c using ZnO as an example. These are (a) the wurtzite (wz; B_4_: with space group $P6_{3}mc$), (b) zinc-blende (zb; B_3_: with space group $F\bar{4}3m$), and (c) the rock salt (rs) NaCl structure (B_1_: with space group Fm3m). The occurrence of diverse crystalline polymorphs depends upon the thermodynamic and environmental growth settings [1].

At ambient conditions, the most thermodynamically secured phase of ZnO is the wz structure. The zb ZnO crystal structure can be stabilized if epitaxially grown on GaAs and/or Si substrates [1,11]. The rs crystalline phase is formed at a relatively high pressure [1]. To exploit the flexibility of III-N and II-O materials in basic physics/chemistry and for their potential use in various applications, it is preferred to consider similar crystalline phases (Figure 1a,c). More recently, the technologically favored zb structures of XOs binary thin films, alloys, and heterostructures have been successfully synthesized [69,70] for their use in practical applications. Therefore, it is important to use empirical models and report the results of a systematic, comparative study for the structural (bond length), elastic, and mechanical properties of the novel zb III-Ns and II-Os.

### 2.2. Bond-Orbital Model

The BOM that we have adopted here is an extension of the method developed earlier by Hall [71] for studying the electronic properties of diamond-type materials, and a generalization of Weaire and Thorpe’s [72] approach for tetrahedrally coordinated binary compounds. This scheme evolved into first-principles theory and has been successfully used in the past to accomplish approximate but meaningful predictions of the bonding properties in crystalline solids [62,63,64,65,66]. A wide range of published literature exists on the merits and limitations of the BOM (see Ref. [63] for a comprehensive review). Calculations exist, ranging from the first-nearest neighbor, orthogonal valence orbitals (s and p), to third nearest-neighbor, d-orbitals, and nonorthogonal sets. Moreover, its connection with the self-consistent density-functional theory has also been explored [68], providing a more fundamental ground to the matrix elements entering into the tight-binding procedure. In terms of LCAO, the formulation of the total energy provides a clear way of assessing the major trends in material characteristics entirely in terms of the atomic orbitals. For tetrahedrally coordinated III-Ns and II-Os, one can choose the orthogonal and normalized sp^3^ hybrids, in which the electron charge density is largest in the direction of the nearest neighbors.

#### 2.2.1. Bond Length and Polarity

Following the ideas of Harrison [63], earlier, Baranowski [62] derived an expression for predicting the equilibrium lattice spacing $d_{0}$ in tetrahedrally coordinated solids by using:

(1) $$d_{0}=\frac{{\left(2\eta_{\sigma }\hbar^{2}/m\right)}^{1/2}}{{\left(k_{ij}^{2}{\bar{\varepsilon }}_{h}^{2}-4V_{3}^{2}\right)}^{1/4}},$$

where, $for\;{sp}^{3}\;bonds,\;the\;term\;\eta_{\sigma }\left(\equiv \frac{1}{4}\eta_{ss\sigma }-\frac{\sqrt{3}}{2}\eta_{sp\sigma }-\frac{3}{4}\eta_{pp\sigma }\right)$ considered [63] the dimensionless universal parameter values of $\eta_{ss\sigma }=-1.40$, $\eta_{sp\sigma }1.84$, $\eta_{pp\sigma }=3.24$, and $\frac{\hbar^{2}}{m}=7.62\;eV{Å}^{2}.$

The weighted average ${\bar{\varepsilon }}_{h}$ of cation–anion hybrid energy is [63]:

(2) $${\bar{\varepsilon }}_{h}=\frac{1}{8}\left(n_{c}\varepsilon_{h}^{c}+n_{a}\varepsilon_{h}^{a}\right),$$

where $n_{c}$ and $n_{a}$ are the numbers of electrons associated with the cation and anion, which participate in the formation of bonds, while $\varepsilon_{h}^{c}$ and $\varepsilon_{h}^{a}$ represent the average values of hybrid cation and anion energies of the sp^3^ bonds [63] requiring free-atomic energies of s and p states for the cation and anion, respectively. The term $V_{3}[\equiv \frac{1}{2}\left(\varepsilon_{h}^{c}-\varepsilon_{h}^{a}\right)]$ in Equation (1) is the hybrid polar energy.

In terms of $V_{3}$ and hybrid covalent energy $V_{2}[\equiv -\eta_{\sigma }\frac{\hbar^{2}}{md^{2}}$], one can obtain the bond polarity,

(3a) $$\alpha_{p}\equiv \frac{V_{3}}{{\left(V_{2}^{2}+V_{3}^{2}\right)}^{\frac{1}{2}}},$$

and covalency,

(3b) $$\alpha_{c}\equiv \frac{\left|V_{2}\right|}{{\left(V_{2}^{2}+V_{3}^{2}\right)}^{\frac{1}{2}}}.$$

In Equation (1), Baranowski [62] used different values of k (≡2.30 C, 1.45 Si, 1.33 Ge, and 1.12 Sn) for elemental semiconductors, which provided a reasonably good result for the bond lengths $d_{0}$, comparable to the experimental data. For compound semiconductors $A_{i}B_{j},$ we have estimated the effective values of $k_{ij}$ ${\left(\equiv k_{i}k_{j}\right)}^{1/2}$ from the appropriate k’s of their respective rows i and j.

#### 2.2.2. Bulk Modulus and Elastic and Shear Constants

For semiconductors and following Baranowski [62], it is possible to calculate the bulk modulus B by using:

(4) $$B=\frac{2}{\sqrt{3}}\left(V_{2}\alpha_{c}^{3}+\frac{7.8}{d^{2}}\right)\frac{1}{d^{3}}.$$

To evaluate the shear elastic constant $C[=\frac{1}{2}\left(c_{11}-c_{12}\right)]$, the author of [62] modified the BOM in the framework of a rigid-hybrid scheme following Harrison [63] and deduced the expression:

(5) $$C=\frac{\sqrt{3}}{4}[V_{2}\alpha_{c}^{3}\left(1+\lambda \right)-\frac{3}{4}\left|V_{pp\pi }\right|\alpha_{c}]\frac{1}{d^{3}},$$

where λ is a dimensionless parameter,

(6) $$\lambda =\frac{-\sqrt{3}V_{sp\sigma }-3V_{pp\sigma }}{V_{ss\sigma }-2\sqrt{3}V_{sp\sigma }-3V_{pp\sigma }}.$$

The terms $V_{sp\sigma }$, $V_{ss\sigma }$, and $V_{pp\sigma }$ are the matrix elements [63]. Combining Equations (4) and (5), one can obtain:

(7) $$c_{11}=\frac{1}{\sqrt{3}}\left[V_{2}\alpha_{c}^{3}\left(3+\lambda \right)-\frac{3}{4}\left|V_{pp\pi }\right|\alpha_{c}+2\frac{7.8}{d^{2}}\right]\frac{1}{d^{3}}$$

and

(8) $$c_{12}=\frac{1}{2\sqrt{3}}\left[V_{2}\alpha_{c}^{3}\left(3-\lambda \right)+\frac{3}{4}\left|V_{pp\pi }\right|\alpha_{c}+4\frac{7.8}{d^{2}}\right]\frac{1}{d^{3}}.$$

In the framework of the VFF model [67], the elastic stiffness constant $c_{44}$ and Kleinman’s internal displacement parameter ζ for the diamond and zb crystal structured materials can be evaluated:

(9) $$c_{44}=\frac{3(c_{11}+2c_{12})\left(c_{11}-c_{12}\right)}{\left(7c_{11}+2c_{12}\right)}$$

and

(10) $$\zeta =\frac{\left(c_{11}+8c_{12}\right)}{\left(7c_{11}+2c_{12}\right)}.$$

### 2.3. Keating Model Parameters: The Force Constants and Elastic Constants

While the VFF [67] and BOM [62,63,64,65,66] have been successfully used for describing the fundamental properties of several elemental (diamond type) and zb binary semiconductors [67], the correctness and reliability of these methods for the ternary alloy systems is hard to assess. An earlier study for the GaInP alloys [68] indicated, however, that the accuracy of the VFF is sufficient to describe and predict the alloy properties, including their bond lengths and phase diagrams. For II-Os, the detailed tests about the applicability of BOM and VFF models and their precision for assessing structural, elastic, and mechanical properties are still not available. Therefore, it is interesting to calculate the bond-stretching α and bond-bending β force constants and evaluate the elastic constants of III-Ns and II-Os using the Keating model [67]. Comparison of BOM results (cf. Section 3) for the elastic constants and mechanical properties with the Keating VFF model will certainly help establish its suitability (cf. Section 2.2). Earlier, Martin [67] obtained the values of α and β for different semiconductors in terms of their macroscopic bulk moduli B using:

(11) $$3B=\frac{\sqrt{3}}{4d}\left(3\alpha +\beta \right)-0.355SC_{0},$$

and the shear constant C:

(12) $$2C=\left(c_{11}-c_{12}\right)=\frac{\sqrt{3}}{d}\beta +0.053SC_{0}.$$

Here, the Keating parameter $SC_{0}$ is a Coulomb contribution to the binary compound semiconductors. The term S is related to the dynamic effective charge, which can be obtained from the optical $\left(\omega_{LO}-\omega_{TO}\right)$ phonon-mode splitting, while $C_{0}\;is\;related\;to\left(=\frac{e^{2}}{d^{4}}\right)$. Using Equations (11) and (12), one can obtain the bond-bending βforce constant:

(13a) $$\beta =\frac{d}{\sqrt{3}}[2C-0.053SC_{0}],$$

and bond-stretching force constant:

(13b) $$\alpha =\frac{4d}{\sqrt{3}}\left[B+0.355\frac{SC_{0}}{3}\right]-\frac{\beta }{3}.$$

Again, in terms of α, β, and the Keating parameter ($SC_{0}$), one can accomplish the following relationships between the elastic constants ($c_{11}$, $c_{12}$, and $c_{44}$):

(14a) $$c_{11}=\frac{\sqrt{3}}{4d}\left(\alpha +3\beta \right)-0.083SC_{0},$$

(14b) $$c_{12}=\frac{\sqrt{3}}{4d}\left(\alpha -\beta \right)-0.136SC_{0},$$

(14c) $$c_{44}=\frac{\sqrt{3}}{4d}\left(\alpha +\beta \right)-0.136SC_{0}-A\xi^{2},$$

where

(14d) $$A=\frac{\sqrt{3}}{4d}\left(\alpha +\beta \right)-0.266SC_{0}$$

and

(14e) $$\xi =\frac{1}{A}\left[\frac{\sqrt{3}}{4d}\left(\alpha -\beta \right)-0.294SC_{0}\right].$$

By using the Equations (14a)–(14e), one can also derive a simple expression for the internal strain parameter, ξ:

(14f) $$\xi =(2c_{12}-C^{\prime })/(c_{12}+c_{12}-C^{\prime }),\;\mathrm{with}\;C^{\prime }=0.314SC_{0}.$$

It is to be noted that the formulas (cf. Equations (14a)–(14c)) for the elastic constants $c_{ij}$ involved only three parameters, of which one, S, is fixed by the optical-phonon mode splitting. Again, by using Equations (14a)–(14f), one can achieve a simple relationship among the elastic constants:

(14g) $$\chi =\frac{2C_{44}(C_{11}+C_{12}-C^{\prime })}{\left(C_{11}-C_{12}\right)\left(C_{11}+{3C}_{12}-{2C}^{\prime }\right)+0.831C^{\prime }(C_{11}+C_{12}-C^{\prime })}=1,$$

which can be used to check the accuracies of the experimental and/or theoretical data for $c_{ij}$. Moreover, the above relationship reduces to the form given by Keating if we set $C^{\prime }$= 0.

### 2.4. Born Transverse Effective Charge

Born effective charge plays a crucial role in the understanding and design of various electronic device structures, particularly those that rely on the polarization effects. For instance, in polar materials, $e_{T}^{*}$ is directly related to impacting (i) How much polarization is generated per unit atomic displacement for providing an insight into the material’s ability to store electrical charge. (ii) In piezoelectric devices, a large $e_{T}^{*}$ indicates strong coupling between the mechanical strain and electrical polarization, leading to an enhanced piezoelectric response. (iii) In ferroelectric memories, it plays a critical role in determining the switching behavior of polarization, as it influences the energy barrier between the different polarization states. (iv) In ionic conductors, like solid-state batteries, the $e_{T}^{*}$ provides information about the ease of ion movement within the crystal lattice, impacting the conductivity and performance of the battery.

Clearly, the understanding of $e_{T}^{*}$ can allow researchers to gain insights into the underlying mechanisms driving polarization in a material, including the role of different atomic species and their bonding characteristics. These efforts may also help in tailoring the material composition and heterostructure to enhance the desired functionalities in an electronic device. As mentioned earlier, the term S in Keating’s VFF model is related to the long-range Coulomb force [67]. In polar semiconductors, it produces the optical phonon splitting $\omega_{LO}$ − $\omega_{TO}$ at the center of the Brillouin zone and can be used in evaluating the macroscopic Born’s transverse effective charge, $e_{T}^{*}$, via the following relationship:

(15) $$S=\frac{{e_{T}^{*}}^{2}}{\varepsilon_{\infty }}=\frac{\mu }{4\pi Ne^{2}}({\omega_{LO}}^{2}-{\omega_{TO}}^{2}),$$

where N is the ion-pair density, μ is the reduced mass of the ion-pair, e is the electron charge, and the term $\frac{1}{\varepsilon_{\infty }}$ rigorously incorporates the screening of macroscopic field by the inter-band electronic transitions [67].

### 2.5. Mechanical Properties

Integrating the VFF [67] and BOM [62,63,64,65,66] models to obtain the elastic constants ($c_{11}$, $c_{12}$, and $c_{44}$) and bulk moduli (B) has offered links between the mechanical and dynamical behaviors of tetrahedrally coordinated materials. In III-N and II-O semiconductors, these values can provide valuable information about the nature of forces operating within the crystals. Next (cf. Section 2.5.1), by using B and $c_{ij}$, we will calculate the shear moduli G, Poisson’s ratio ν, Young’s moduli Y, and microhardness H. The comparison of our predictions for the mechanical properties of III-Ns and II-Os with first-principles calculations would certainly help understand the accuracy of our approach for evaluating the materials’ response to the application of high pressure.

#### 2.5.1. Shear Moduli, Poisson’s Ratio, Young’s Moduli, and Microhardness

Although the bulk modulus B is related to $c_{11}$ and $c_{12}$, there exist no clear expressions relating a polycrystal averaged shear moduli G to the elastic constants $c_{ij}$. By using different methodologies [73,74], approximate averages for the lower and upper bounds of G have been suggested. While Voigt [73] derived an expression for the upper bound:

(16a) $$G_{V}=\frac{\left(c_{11}-c_{12}+3c_{44}\right)}{5},$$

Reuss and Angew [74] provided a relationship for estimating the lower bound:

(16b) $$G_{R}=\frac{5(c_{11}-c_{12})c_{44}}{4c_{44}+3(c_{11}-c_{12})}.$$

An arithmetic average of the Voigt [73] and Reuss and Angew [74] methods was used here for evaluating the average shear modulus: $G=\frac{1}{2}(G_{V}+G_{R})$. Other important mechanical parameters related to B and G include the Poisson’s ratio:

(17a) $$\nu =\frac{3B-2G}{2\left(3B+G\right)\;\;\;\;},$$

Young’s modulus:

(17b) $$Y=\frac{9BG}{3B+G},$$

and the microhardness:

(17c) $$H=\frac{(1-2\nu )Y}{6(1+\nu )}.$$

By integrating BOM and VFF models, we have systematically assessed the structural, elastic, and mechanical characteristics of zb III-Ns and II-Os. The results reported in Section 3 include the bond length d, bond polarity $\alpha_{P}$, covalency $\alpha_{C}$, elastic constants, bulk moduli B, bond-stretching α and bond-bending β force constants, Born’s transverse effective charge $e_{T}^{*}$, etc. Internal displacement parameters ζ and Poisson’s ratios ν are used to link the mechanical parameters (viz., Young’s modulus Y, shear modulus G, and microhardness H) with the bond length d. The results are analyzed by comparing/contrasting with the existing theoretical and experimental data, wherever available.

## 3. Numerical Computations, Results, and Discussion

The increasing demands for greater flexibility in the emission wavelengths and improved device performance with reduced costs are directing the search for novel optoelectronic materials away from the traditional binary III–V and III–N semiconductors and their ternary and quaternary solid solutions. Currently, many devices are being designed using binary Be, Mg, Zn, Cd, oxides, and their related alloys. These materials have generated considerable interest, as they could provide, in principle, an accessible direct WBG ranging from ~2.3 eV (539 nm) CdO to ~7.7 eV (161 nm) MgO. Such choices have made II-Os promising candidates even for deep ultraviolet lighting applications. Despite many first-principles DFT calculations of the structural and electronic characteristics [30,31,32,33,34,35,49,50,51,52,53,54,55,56,57,58,59] based on LDA by implementing the relativistic linearized augmented plane wave (LAPW) method, very few experimental efforts have been made measuring the equilibrium bond lengths d in the zb II-O semiconductors. Several first-principles calculations have reported the values of d either for the rs and/or the wz structured materials. For the zb and/or wz crystals, however, the LDA calculations [49,50,51,52,53,54,55,56,57,58,59] have generally underestimated d by 2 to 5%.

### 3.1. Bond Length

Using the BOM (cf. Section 2.1), we compared (see Table 1) the values of bond lengths d for the zb III-Ns and II-Os with the existing experimental and first-principles calculations [30,31,32,33,34,35,49,50,51,52,53,54,55,56,57,58,59]. For the zb III-Ns, the comparisons made between our calculations and the experimental values of d were quite encouraging, providing an accuracy of a few (≤5.11%) percent. For II-Os, however, the model revealed relatively large discrepancies in d values when a comparison of the BOM results was made against the limited data from the experimental and/or theoretical calculations, with the worst divergences noticed in BeO and ZnO (<12%).

By applying the BOM on more then 40 different tetrahedrally coordinated semiconductors, we have noticed that the model made a reasonably good prediction (<6%) for the equilibrium spacing d in many iso-row III–V, II–VI, and I–VII binary materials with the column IV elemental semiconductors. For instance, the values of d for GaAs, ZnSe, and CuBr were nearly the same as that of Ge (<1%), AlP was in line with Si, and InSb, CdTe, and AgI exhibited good agreement with the bond length of Sn. While the bond length of the BN was closer to C (<4%), the d value of iso-row BeO, however, differed from BN and C by ~12%.

One must note that the orbital interactions required for determining the bond length d values in different materials are extremely complicated. Based on the physics [63] of chemical bonding, our intuitive observation has suggested that an oversimplified BOM cannot predict accurate values of d for all the iso-row semiconductors, especially the metal carbides and oxides, which form different polymorphs [1]. The discrepancies in such materials can arise from several factors, including an oversimplification of the orbital overlap, neglecting electron correlation and the limitations in the hybridization scheme used. Choosing a suitable basis set for calculations can possibly improve the accuracy of bond length predictions. Thus, our choice, where only the sp^3^ orbitals were considered, could provide improvement by including d-orbitals [27]. Moreover, we strongly feel that additional experimental efforts must also be made for extracting accurate data on d values for the zb II-Os.

### 3.2. Bond Polarity

The zb III-N (II-O) materials have shown significant bond polarity due to large electronegativity differences between the group III (II) elements and nitrogen N (O) atoms. Thus, a III-N (II-O) bond indicates a partial positive charge on group III (II) atoms, with a negative charge on the N (O) atoms. Compound semiconductors are non-centrosymmetric, which means that the stacking of atoms along certain crystallographic orientations is not symmetric. In zb materials, the [111] is a polar direction, with separation of charges along this direction for producing an internal electric field and hence the polarity. This is especially important for III-Ns (II-Os) where the internal electric field is large. As the epitaxial layers in designing different device structures are generally grown along the polar direction, it is important to estimate the bond polarity $\alpha_{p}$ in such technologically important zb materials.

#### Links of $\alpha_{p}$ with $f_{i}$

Earlier, Van Vechten [75], Phillips [76], and Pauling [77] had studied the relationships between the chemical properties of II–VI, III–V, and I–VII crystals by calculating the electronic band structures. Their concept evolved from the molecular picture in terms of the bonding and antibonding states separated by the energy bandgaps $E_{g}$. This notion allowed them [75,76,77] to designate chemical trends in a large family of crystals by using only a few parameters for estimating the bond ionicity $f_{i}$ (or bond covalency). In Table 2, we have listed our BOM results of the polarity $\alpha_{p}\left(\equiv f_{i}^{1/2}\right)$ [78] for III-Ns and II-Os and compared them with the calculations of ionicity reported by Philips [76], Pauling [77], Coulson et al. [79], Garcia et al. [80], and Christensen et al. [81,82]. Based on the simplicity of our model, the predictions of bond-ionicity for the zb III-N materials have agreed reasonably well with the published data [75,76,77,78,79,80]. Once again, the discrepancies in $\alpha_{p}$ (or $f_{i}$) for II-Os (see Table 2) are related to the lack of experimental and/or reliable theoretical data for the zb structures.

### 3.3. Elastic Constants, Bulk Moduli, and Bond-Stretching and Bond-Bending Force Constants

For the zb III-Ns and II-Os, we have reported in Table 3 the results of BOM calculations for the covalency $\alpha_{c}$, bulk modulus ${B[=(c}_{11}+2c_{12})/2]$, stiffness $C=[c_{11}-c_{12})/2]$ (Equation (12)), and elastic stiffness constant $c_{44}$ (10^11^ dyn/cm^2^). Bond-stretching α (Equation (13b)) and bond-bending β (Equation (13a)) force constants (10^3^ dyn/cm) are also listed along with the ratios of κ $\left(=\frac{C}{B}\right),$ $c_{12}/c_{11}$, β/α, Kleinman’s internal displacement parameter ζ (Equation (10)), and an accuracy check χ for the $c_{ij}$ (Equation (14g)).

The calculations for III-N and II-O materials provided strong confirmations to the Born criteria for satisfying the three necessary and sufficient conditions $\left(i.e.,\;(c_{11}-c_{12}\right)$ > 0, $\left(c_{11}+2c_{12}\right)$ > 0, and $c_{44}$ > 0), which indicate that all the binary compounds were mechanically stable. From Table 3, one can also notice that the values of B, C, $c_{44},$ α, and β consistently decreased as one moved from B → Al → Ga → In (Be → Mg → Zn → Cd) in III-Ns (II-Os). It is to be noted that ζ describes the relative position of cation and anion atoms on the zb sublattices due to the volume-conserving strains. In the tetrahedrally coordinated materials, it caused a trigonal distortion along the [111] direction. Obviously, the smaller value of ζ implies a larger resistance against the bond-angle distortions, while the reverse is true with the higher values. For III-Ns, the reliable values of ζ reported by Shimada et al. [83] by using first-principles MD simulations compared reasonably well with the BOM calculations. The structural and elastic constants obtained from BOM and VFF models (see Table 3) for III-Ns were found in very good agreement with the experimental [2] as well as converged first-principles calculations [30,31].

**Figure 2:**
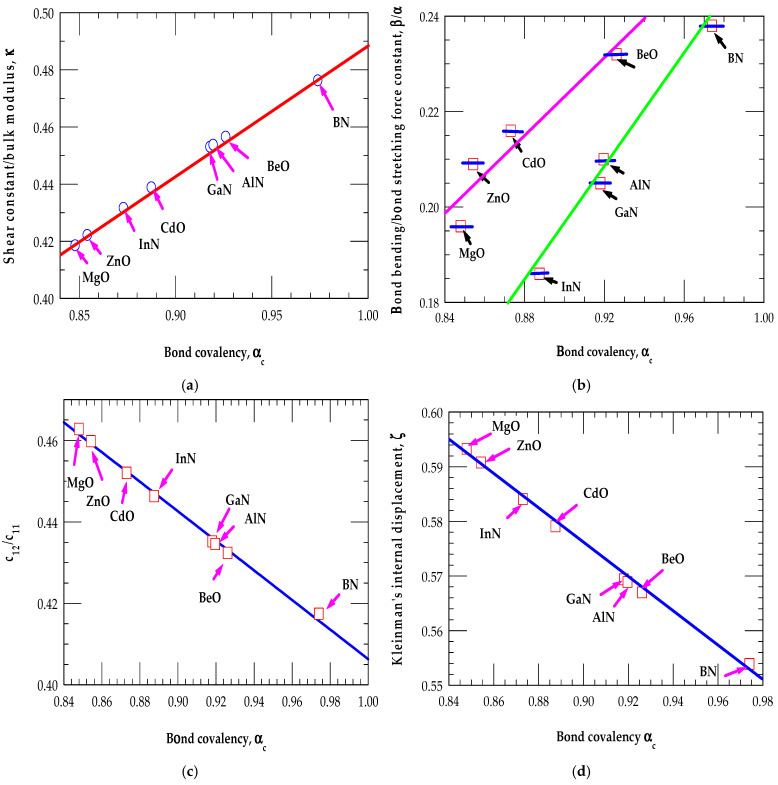
The BOM results for different physical parameters as a function of the bond covalency $\alpha_{c}$ in partially covalent III-Ns and partially ionic II-O materials, revealing interesting trends: (**a**) the value of κ $\left(=\frac{C}{B}\right)\;increased\;with\;an\;increase\;of\;\alpha_{c},$ (**b**) the ratio of $\frac{\beta }{\alpha }\;increased\;with\;an\;increase\;of\;\alpha_{c}$, (**c**) the elastic constant ratio $\frac{c_{12}}{c_{11}}\;decreased\;with\;an\;increase\;of\;\alpha_{c}$, and (**d**) the Kleinman’s displacement ζ $decreased\;with\;an\;increase\;in\;\alpha_{c}$ (see the main text).

From the simulated structural and elastic properties, one may notice (see Figure 2a,d) that AlN and GaN (MgO, ZnO) materials exhibited similar characteristics, while BN (BeO) was considerably harder than InN (CdO), which revealed significantly softer bonding. The Keating’s bond-bending force constant β in BN (BeO) displayed a trend like that of the crystalline diamond (C). Relatively large but significantly different values of β/α have been noticed for the partially covalent III-Ns and partially ionic II-Os. These results are consistent with the higher covalency $\;\alpha_{c}$ or lower Kleinman’s displacement ζ parameters.

Moreover, our calculated ratios of $\frac{c_{12}}{c_{11}},\kappa \;\left(=\frac{C}{B}\right),$ β/α, and ζ for the partially covalent III-Ns and partially ionic II-Os revealed fascinating links with the bond covalency $\alpha_{c}.\;$Clearly, the calculations of different quantities, such as κ $\left(=\frac{C}{B}\right),$ β/α, $\frac{c_{12}}{c_{11}},and\;$ ζ indicated a steady increase (Figure 2a,b) and decrease (Figure 2c,d) with the increase in covalency $\alpha_{c},$ respectively. These trends, noticed earlier in different semiconductor materials by Martin [67], Kitamura et al. [60,61], Harrison [63], and Falter et al. [78], are consistent with our BOM and VFF results for III-Ns and II-Os.

Again, in most semiconductors, the ratio of bond-bending to bond-stretching force constants β/α is typically less than <1 (see Table 3). This means that the resistance to the bond-stretching force constant is customarily greater than the resistance to the bond-bending force constant. It can also be related to a general perception that the bonding forces in semiconductors are predominantly related to stretching of the covalent bonds along the interatomic axis, making it energetically more costly for significantly changing the bond length, as compared to the bond angle. The exact ratio of β/α can vary depending upon the specific material, its crystal structure, and the bonding characteristics (i.e., from covalent (IV–IV) → partially covalent (III–V) → partially ionic (II–VI) → ionic (I–VII)). A smaller ratio of β/α indicates that it takes a larger force to stretch a bond than to bend it, reinforcing the directional nature of the covalent bonds in semiconductors.

### 3.4. Born’s Transverse Effective Charge

In binary polar materials, Born’s transverse effective charge $e_{T}^{*}$ is defined as a variation of the force on a given atom under the applied electric field. It is the key parameter used for understanding the coupling between the lattice vibrations and the electric field. In most simple diatomic crystals, including the III–V, II–VI, and I–VII semiconductors of zb and wz structures, the frequencies of long-wavelength transverse optical (TO; $\omega_{TO}$) and longitudinal optical (LO; $\omega_{LO}$) modes are known (see Table 4), along with the low- and high-frequency dielectric constants ($\varepsilon_{0}$ and $\varepsilon_{\infty }$).

In these crystals, the oscillator strength of the TO phonon frequency is reflected in the difference between the squares of their LO and TO modes or, alternatively, via the Lyddane–Sachs–Teller relationship $\left(\frac{\omega_{LO}^{2}}{\omega_{TO}^{2}}=\frac{\varepsilon_{0}}{\varepsilon_{\infty }}\right).$ Thus, the oscillator strength is commonly described by one of the two effective charge parameters, $e_{T}^{*}$ or$\;e_{S}^{*}[=3e_{T}^{*}/\varepsilon_{\infty }+2]$—the so-called Szigeti effective charge. Out of these two charge parameters, the macroscopic effective charge $e_{T}^{*}$ is model independent and can be calculated from the readily observable quantities. On the other hand, the definition of the derived parameter describing $e_{S}^{*}$ at a specific position in a crystal is model-dependent, requiring specific assumptions on the form of the effective field.

Except for III-Ns, the optical phonon frequencies of zb II-Os have not been measured experimentally by using either the far-infrared and/or Raman scattering spectroscopy. Here, we used Equation (15) and calculated $e_{T}^{*}\;$by incorporating the appropriate theoretical values of $\omega_{LO}$, $\omega_{TO}$, and $\varepsilon_{\infty }$ listed in Table 4. The calculated results, along with Harrison’s effective charge $Z^{*}$ [63], are reported (cf. Table 4) and compared with the existing experimental and theoretical data. Moreover, this study has provided support to the VFF model [67] by confirming the relationship of Keating’s parameter S to $e_{T}^{*}$.

### 3.5. Hardness of Materials

Among the different mechanical properties, microhardness H is the most elementary and practical indication for characterizing the materials’ resistance to the local elastic and plastic deformations [84,85,86,87,88,89,90,91,92,93,94,95,96,97,98,99,100]. Although extensively studied, it has been and remains quite a challenging issue for both material scientists and engineers for achieving a formal theoretical description. Over the past few years, several semiempirical models have been proposed estimating H for different materials based on (a) their bond length, charge density, and ionicity, (b) the strength of chemical bonds, (c) the thermodynamical concept of energy density per chemical bonding, (d) linking it to the materials’ electronegativity, and (e) using a temperature-dependent constraint theory for hardness of multicomponent bulk metallic glasses [84,85,86,87,88,89,90,91,92,93,94,95,96,97,98,99,100].

Experimentally, the microhardness H can be measured by pressing an indenter onto the crystal’s surface and gauging the size of the impression. The reproducibility relationship with the load to the area or depth of the indentation can be used for assessing H. In the semiconductor industry, the type of hardness on different materials (e.g., bulk, thin films, etc.) has been achieved by considering various testers, including a recently developed nano-indentation tester [84]. For different samples, the value of H obtained by using a diamond indenter with the shape of an inverted pyramid is classified as the Vicker’s or Knoop’s microhardness. Since Knoop’s scale incorporates a sharper diamond wedge, its value for the same load is generally lower by at least 10% than Vicker’s scale [84]. Obviously, the microhardness is certainly not a quantity that can be easily determined on a well-defined absolute scale. Different factors, as mentioned above, can add a huge complexity to the formal theoretical definitions of hardness. Again, for many materials, the published data on H [84,85,86,87,88,89,90,91,92,93,94,95,96,97,98,99,100] are generally reported at RT; however, the methods can be applied at higher T up to their melting points.

The relationship between the microhardness and modulus of elasticity has a physical reason, as both the quantities depend on the materials’ structure and the corresponding intramolecular/intermolecular interactions. Due to the intricacies involved in measuring the elastic characteristics of materials, many researchers have, however, preferred simulating them by using different computational techniques. Various correlations between H, Y, and $c_{ij}$ are identified and discussed for both the ionic and covalent solids [85,86,87,88,89,90,91,92,93,94,95,96,97,99,100,101,102,103]. While the resistance to the volume change in a material is linked to the bulk modulus B, the resistance to the change of the shape is connected to the shear modulus G. Moreover, the ratio B/G provides the information for the ductile or brittle behavior [1]. In semiconductors, the Young’s modulus Y and Poisson’s ratio ν have indicated the stiffness and degree of directionality for the covalent bonds, respectively. It has been recognized that the hardness of strongly bonded covalent/ionic crystals is associated directly with the bond strength. Materials with high hardness are technologically important for cutting tools and wear-resistant coatings.

#### 3.5.1. Shear Modulus, Microhardness, and Young’s Modulus

Historically, the identification of a hard and/or a ductile material has been made using different models by associating their mechanical properties. Such naïve empirical correlations have prompted misleading results [97]. Even though Chen et al. [101] elegantly modeled the microhardness of a material by incorporating Pugh’s ratio [102], which characterizes the brittleness/ductility, such a macroscopic concept cannot provide a deeper insight into the physical origin of hardness. By using the methodology outlined in Section 2.4, we have reported in Table 5 our results of the BOM calculations for the shear modulus G, ratio B/G, ν, Y, and H for the zb III-Ns and II-Os. A comparison has been made with the existing theoretical and/or limited measured data [41,85,86,87,88,89,90,91,92,93,94,95,96,97,98,99,100]. In the absence of experimental and/or calculated outcomes for the mechanical characteristics of II-Os, our predictions for the III-N materials compared reasonably well (see Table 5) with the existing microscopic and/or empirical simulations [85,86,87,88,89,90,91,92,93,94,95,96,97,98,99,100].

#### 3.5.2. Variations in Microhardness, Young’s Modulus, and Shear Modulus with Bond Length

**Figure 3:**
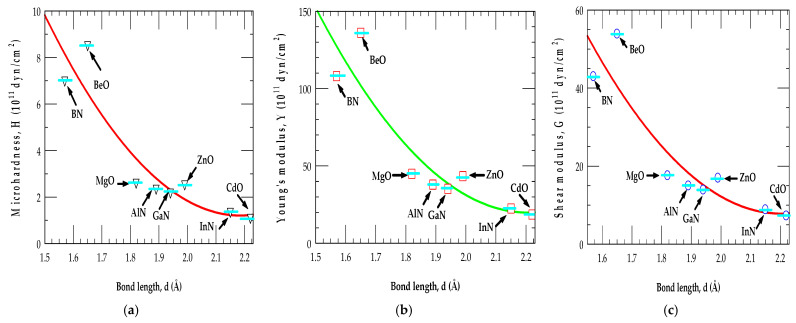
BOM results of mechanical properties shown with error bars (sky-blue-color line) for zb III-Ns and II-Os as a function of bond length, d: (**a**) for microhardness H, (**b**) for Young’s modulus Y, and (**c**) for shear modulus G. Obviously, in these semiconductors, our results have clearly shown a direct relationship between bond length d and mechanical traits, exhibiting an inverse relationship, meaning that as the bond length increased, the mechanical properties tended to decrease. The materials with shorter bonds typically led to higher microhardness H, Young’s modulus Y, and shear modulus G due to stronger interatomic forces within the crystal lattice.

For III-Ns and II-Os, our results of the major mechanical characteristics (e.g., H, Y, and G) have indicated interesting trends with the bond length d: (a) The values of G, Y, and H steadily decreased with the increase of the bond lengths, from BN → AlN → GaN → InN (BeO → MgO → ZnO → CdO), exhibiting nearly identical plots (see Figure 3a–c). (b) Only the BN (BeO) has satisfied the criteria [85,86,87,88,89,90,91,92,93,94,95,96,97,98,99,100] of being a super-hard material (H > 4 (10^11^ dyn/cm^2^)), while the other compounds, AlN and GaN (MgO and ZnO) revealed nearly identical but strong values of H (Y,G), as compared to the weak H (Y,G) for an InN (CdO) material of a longer d. (c) The poisson’s ratio of BN (BeO) is found lower than the other materials.

Obviously, in the III-N and II-O semiconductor materials, our BOM results have shown a direct relationship between their bond lengths and the mechanical properties (including the microhardness H, Young’s modulus Y, as well as the shear modulus G). Obviously, the results (see Figure 3a–c) have indicated that the stronger bonds due to shorter bond lengths can provide greater resistance to the deformation, resulting in the higher values of H, Y, and G.

#### 3.5.3. Variations in Microhardness, Young’s Modulus, and Shear Modulus

**Figure 4:**
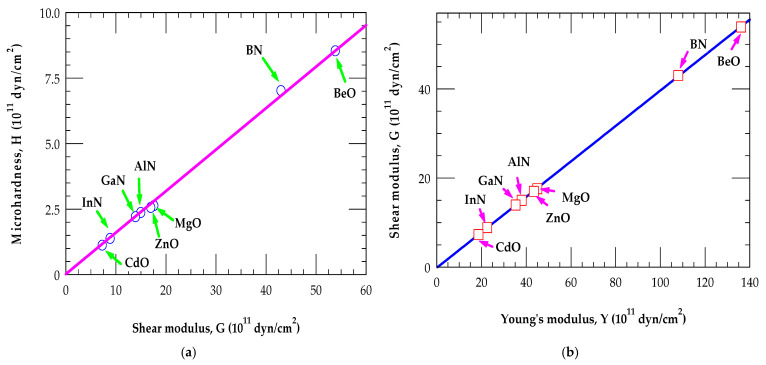
The calculated results of BOM simulations indicating a linear relationship between (**a**) the microhardness H and shear modulus G and (**b**) the shear modulus G and Young’s modulus Y for zb III-N and II-O materials with the single proportionality constants of ~0.16 and ~0.41, respectively (see the main text).

In semiconductors, the microhardness H is different from the shear modulus G. However, these quantities in III-Ns and II-Os can be correlated (see Figure 4a,b)to one another via their physical and chemical characteristics (such as G, Y, and H), as the compressibility and hardness are mechanical responses of the atoms to applied pressure in the closely packed crystals. 

 Yes, H and G are complex properties, related to the extent of how different materials resist the elastic and plastic deformations. Several proposals have been made by different researchers—some have used, however, unrealistic empirical models relating H, G and G, Y [97]. While investigating the mechanical properties of III-N and II-O materials by using BOM, our study has clearly noticed the linear correlations between H, G (see Figure 4a) and G, Y (see Figure 4b), respectively, with single proportionality constants of ~0.16 and ~0.41, suggesting that these mechanical characteristics are certainly not complex, as many empirical models have indicated [85,86,87,88,89,90,91,92,93,94,95,96,97,98,99,100]. The linearity of H/G was observed earlier in a diverse set of materials, including ceramics, intermetallic, metallic glasses, bio-composites, molecular crystals, etc. [97]. We strongly feel that more efforts should be made using the basic chemical and physical properties of different crystalline structured materials for correlating their elastic and mechanical properties.

## 4. Concluding Remarks

Exceptional structural, electrical, chemical, and mechanical properties of novel wide-bandgap III-N and II-O materials have unequivocally demonstrated their versatility across different areas of research in designing optoelectronics, short-wavelength light-emitting diodes, laser diodes, biosensors, and photodetectors to achieve flexible high-power radio-frequency modules, and heat-resistant optical switches for communication networks [1,2,3,4,5,6,7,8,9,10,11,12]. The knowledge of elastic, structural, bonding, and mechanical properties of these materials plays crucial roles for gaining not only the basic understanding between them but also assessing their use in thermal management devices [1]. Due to the large electronegativity difference between N (O) and atoms of III (II) elements, although the chemical bonding in the WBG III-N (II-O) materials is heteropolar, the nature of their quantitative degree of ionicity remains, however, an imprecise concept. By carefully integrating the BOM [62,63,64,65,66] using sp^3^ orbitals with a VFF model [67], we have reported results of a systematic and comparative study for the structural, elastic, bonding, and mechanical properties. For partially covalent III-Ns, the predicted values of bond length d, bond polarity $\alpha_{P}$, covalency $\alpha_{C}$, bulk modulus B, elastic stiffness $C(=\frac{\left[c_{11}-c_{12}\right]}{2})$, bond-stretching α and bond-bending β force constants, Kleinmann’s internal displacement parameter ζ, and Born’s transverse effective charge $e_{T}^{*}$ were all shown to be in very good agreement with several sophisticated calculations [30,31,32,33,34,35,49,50,51,52,53,54,55,56,57,58,59] and the experimental data [68].

Due to limited experimental measurements and first-principles calculations in the partially ionic zb II-Os, our results of their structural, bonding, elastic, and mechanical traits have revealed interesting and comparable trends with the III-Ns [104]. The large discrepancies noticed for the d values (≤12%) in zb BeO and ZnO materials could possibly arise from an oversimplified construction of the BOM, where only the sp^3^ hybrids were considered. The inclusion of d-orbitals in the basis set [27] might improve the calculations for achieving a better accuracy in their bond lengths. We also strongly feel that additional experimental efforts must be made for extracting accurate data for the d values in zb II-Os. In BN (BeO), our calculation of the bond-bending force constant β exhibited a feature like that of the crystalline diamond (C) [63]. Relatively large but different values for β/α were noticed in the partially covalent III-N materials, as compared to the partially ionic II-Os. Again, our systematic calculations of major mechanical characteristics (viz., the shear modulus G, Young’s modulus Y, and microhardness H) have indicated a direct correlation with the bond lengths d: they were all seen steadily decreasing with the increase in d as one moved from BN → AlN → GaN → InN (BeO → MgO → ZnO → CdO). Consistent with the first-principles calculations, our study also showed G, H, and Y exhibiting nearly identical nonlinear declining behavior with the increase in bond length d. Moreover, only the BN (BeO) satisfied the criteria [85,86,87,88,89,90,91,92,93,94,95,96,97,98,99,100] to remain a super-hard (H > 4 (10^11^ dyn/cm^2^)) material [97]. The other compounds, AlN and GaN (MgO and ZnO), revealed nearly identical but strong H (Y,G) values compared to the softer bonding for the InN (CdO) compound due to a longer d. In semiconductors, many empirical relationships have been proposed between their mechanical characteristics, $H\;and\;G\left(or\;B\right)\;$and $G\left(or\;B\right)\;and\;Y—$some of them being quite unrealistic. Using a physicochemical theory, Hu and Yu [97] have recently predicted linear relationships between H and B for the IV–IV, III–V, II–VI, and I–VII crystals, with different slopes of 0.208, 0.172, 0.019, and 0.015, respectively. Our study for the III-Ns and II-Os provided strong corroboration to the results reported in Ref. [97] by achieving linear dependencies between the values of H and G (and G and Y). However, our results predicted a single slope of ~0.16 (and ~0.41) for both the nitrides and oxides. We hope that the projected results with important trends in the structural, elastic, bonding, and mechanical characteristics of the novel III-N and II-O materials will encourage experimentalists to perform similar measurements to check our theoretical conjectures. Based on the super-hard mechanical characteristics of BN (BeO), the material could play a vital role in the electronics, aerospace, defense, nuclear reactors, and automotive industries for providing integrity and performance at higher temperatures in high-power applications, ranging from heat sinks to electronic substrates to insulators in the high-power electronic devices.

## Figures and Tables

**Table 1:** Predicted values of bond lengths of zinc-blende III-N and II-O semiconductors. The experimental data of bond lengths are also reported for comparison with BOM and other calculations.

Material	$d_{0}\left(Å\right)$ (Our) BOM	$d_{0}(Å)$ Experimental	Percentage (%) Error	$d_{0}(Å)$ Others
BN	1.58	1.57	0.64	1.56, 1.54–1.67 ^(a)^, 1.585–1.602 ^(d)^
AlN	1.88	1.89 ^(b)^	0.53	1.913–1.95 ^(c)^, 1.927–1.934 ^(e)^, 1.944 ^(f)^
GaN	1.91	1.94 ^(b)^	1.55	1.983–2.02 ^(c)^, 1.913–2.026 ^(d)^
InN	2.04	2.15 ^(b)^	5.11	2.21–2.26 ^(c)^, 2,22–2.295 ^(g)^
BeO	1.46	1.65	11.5	1.69 ^(j)^,1.65–1.70 ^(k)^, 1.40 ^(n)^
MgO	1.73	1.82	4.95	2.022 ^(h)^, 2.023, 1.75 ^(n)^
ZnO	1.75	1.99	12.0	2.00 ^(h)^, 2.059 ^(i)^, 2.07 ^(m)^, 1.98 ^(n)^
CdO	2.10	2.22	5.4	2.232 ^(h)^, 2.28 ^(l)^, 2.24 ^(m)^

^(a)^ Ref. [31] and references cited there in, ^(b)^ Ref. [68], ^(c)^ Ref. [41], ^(d)^ Refs. [31,41], ^(e)^ Ref. [24], ^(f)^ Ref. [47], ^(g)^ Ref. [54], ^(h)^ Ref. [26], ^(i)^ Ref. [15], ^(j)^ Ref. [20], ^(k)^ Ref. [19], ^(l)^ Ref. [14], ^(m)^ Ref. [12], and ^(n)^ Ref. [25].

**Table 2:** Comparison of the BOM calculations (using Equation (3a)) for the bond polarity $\alpha_{p}$ of the zinc-blende III-N and II-O semiconductors with other calculations reported in the literature for zb, wz, and rs structures.

Material	BOM	f_Ph_: Phillips ^(a)^	f_P_: Pauling ^(b)^	f_Co_: Coulson ^(c)^	f_G_: Garcia ^(d)^	f_C_: Christensen ^(e)^	Ref. [78] Calc.
BN	0.227	0.221	0.22	0.35	0.484	0.380	
AlN	0.393	0.449	0.43	0.36	0.754	0.775	
GaN	0.393	0.500	0.39	0.36	0.778	0.770	
InN	0.461	0.578	0.34	0.36	0.853	0.859	
BeO	0.377	0.602 ^(wz)^	0.63 ^(wz)^	0.64 ^(wz)^			0.61 ^(wz)^
MgO	0.530	0.841^(rs)^	0.73 ^(rs)^				0.88 ^(rs)^
ZnO	0.520	0.61 ^(wz)^	0.59 ^(wz)^	0.65 ^(wz)^			0.69 ^(wz)^
CdO	0.488	0.785 ^(rs)^	0.55–0.85 ^(rs)^				

^(a)^ Ref. [76], ^(b)^ Ref. [77], ^(c)^ Ref. [79], ^(d)^ Ref. [80], ^(e)^ Refs. [81,82], ^(wz)^ wurtzite, and ^(rs)^ rock salt.

**Table 3:** Using BOM and Martin’s VFF models, we report the results of our calculations for the elastic constants (using Equations (7)–(9) and (14a)–(14c)), bulk modulus, ${B[=(c}_{11}+2c_{12})/2]$, ${C=[(c}_{11}-c_{12})/2]$ (Equation (12)), $c_{44}\;BOM\;and\;{VFF}^{\left(a\right)}$(10^11^ dyn/cm^2^), the ratios of $c_{12}/c_{11}$ and $\kappa \left(=\frac{C}{B}\right),$ bond-stretching α and bond-bending β force constants (10^3^ erg/cm^2^; Equations (13a) and (13b)), the ratio β/α, Kleinman’s internal displacement parameter ζ (using Equation (10)), and the prediction of elastic constants using Equation (14g) for zb III-Ns and II-Os.

Material	$\alpha_{c}$	B	C	$c_{44}$	κ	$c_{12}/c_{11}$	ζ	α	β	β/α	χ
BN	0.97	73.67	35.08	49.3, 59.19 ^(a)^	0.48	0.42	0.55, 0.10–0.28 ^(b)^	261.54	62.20	0.24,0.26–0.40 ^(b)^	1.0007
AlN	0.92	26.81	12.14	17.31,22.2 ^(a)^	0.45	0.44	0.57, 0.56–0.60 ^(b)^	121.10	24.80	0.21, 0.15 ^(b)^	1.0015
GaN	0.92	24.86	11.28	16.07,20.3 ^(a)^	0.45	0.44	0.57, 0.50–0.67 ^(b)^	112.45	23.58	0.21, 0.15 ^(b)^	1.0013
InN	0.89	16.28	7.144	10.28,13.76 ^(a)^	0.44	0.45	0.58, 0.70 ^(b)^	83.38	15.50	0.18, 0.09 ^(b)^	1.0019
BeO	0.93	95.57	43.63	62.07, 73.36 ^(a)^	0.46	0.43	0.57	310.97	72.12	0.23	1.0006
MgO	0.85	33.56	14.05	20.48,25.58 ^(a)^	0.42	0.46	0.59	136.70	26.77	0.20	1.0013
ZnO	0.85	32.20	13.59	19.76, 23.63 ^(a)^	0.42	0.46	0.59	127.88	26.71	0.21	1.0008
CdO	0.87	13.58	5.86	8.47, 10.07 ^(a)^	0.43	0.45	0.58	64.18	13.88	0.22	1.0007

^(a)^ Ref. [67] and ^(b)^ Refs. [31,83].

**Table 4:** For zinc-blende III-Ns and II-Os, we have listed the experimental results of $\omega_{LO}$ and $\omega_{TO}$ for the optical phonons and their frequency splitting ∆ω (in cm^−1^), along with the high-frequency dielectric constants. Using Equation (15), the calculated results of $e_{T}^{*}$ are reported, along with Harrison’s effective charge $Z^{*}$ [63]. Comparison is also made with the existing experimental and/or theoretical data [31]. Our calculation of the Keating’s parameter S relating to $e_{T}^{*}\;$in Equation (15) has provided support to the VFF model [67] (see the main text).

Materials	$\omega_{LO}$	$\omega_{TO}$	∆ω	$\varepsilon_{\infty }$	S	Others ^(a)^	$Z^{*}$ ^(b)^	Others ^(a)^	$e_{T}^{*}$	Others ^(a)^
BN	1305	1054	251	4.46	0.866	0.858	0.482	0.36	1.97	1.87–1.93, 2.00–2.47
AlN	907	662	245	4.68	1.478	1.551	1.458	1.33	2.70	2.52–2.56, 2.36–2.75, 3.20
GaN	743	552	191	5.35	1.325	1.246	1.458	1.43	2.66	2.59–2.68, 2.50, 2.43–3.20
InN	556	467	89	5.50	2.015	1.983	1.812	1.76	2.42	2.73, 3.10
BeO	1074	721	353	3.10	1.028	-	0.375	0.55	1.79	1.12–1.83
MgO	709	430	279	3.16	1.155	-	1.136	-	1.91	1.77
ZnO	558	403	155	5.32	0.948	-	1.092	1.15	2.18	1.95–2.09
CdO	411	335	76	7.20	0.546	-	0.943	-	1.98	-

^(a)^ Ref. [31] and ^(b)^ Ref. [63].

**Table 5:** Comparison of the calculated mechanical properties of partially covalent III-Ns and partially ionic II-Os using the BOM. The results of shear modulus G, B/G, Poisson’s ratio ν, Young’s modulus Y, and microhardness H are compared with existing experimental and theoretical data.

Mechanical Properties							
Material	G (Our, Others)	B/G (Our, Others)	ν (Our, Others)	Y (Our)	Y (Others)	H (Our)	H (Others)
BN	43.02, 40.34 ^(a)^	1.71	0.25, 0.13–0.21 ^(a)^	108.02	99.8–107.0 ^(d)^, 94.06 ^(e)^,90.6 ^(f)^	7.01	4.8,7.41 ^(j)^, 6.2–7.14 ^(j)^
AlN	15.02, 12.21 ^(a)^	1.79, 1.54 ^(b)^	0.26, 0.24 ^(a)^	37.96	30–35 ^(g)^, 30.19 ^(e)^, 32.3 ^(f)^	2.36	2.07 ^(j)^, 1.39–1.8 ^(j)^
GaN	13.95	1.783, 1.60 ^(b)^	0.26, 0.26 ^(a)^	35.25	26–29, 28.5 ^(g)^,30.2	2.20	1.41–1.51 ^(j)^, 1.74–1.94 ^(k)^
InN	8.88	1.833	0.27, 0.31 ^(a)^	22.55	14.9 ^(i)^, 20–25 ^(h)^, 23.107 ^(f)^	1.37	1.23 ^(k)^, 1.25 ^(f)^, 0.5–0.88 ^(j)^
BeO	53.89	1.773	0.26	136.1	37.2 ^(j)^	8.53	
MgO	17.61	1.91, 1.42–1.54 ^(c)^	0.28	44.96		2.62	2.48 ^(g)^
ZnO	17.006	1.894	0.28	43.38	11.1 ^(k)^	2.55	
CdO	7.307	1.858	0.27	18.59		1.11	

^(a)^ Refs. [86,93], ^(b)^ Ref. [92], ^(c)^ Ref. [91], ^(d)^ Ref. [85], ^(e)^ Ref. [86], ^(f)^ Ref. [87], ^(g)^ Ref. [41], ^(h)^ Ref. [88], ^(i)^ Ref. [89], ^(j)^ Refs. [94,97], and ^(k)^ Ref. [90].

## Data Availability

The data that support the findings of this study will be available from the corresponding author upon reasonable request due to privacy.
